# Improving Sleep Quality and Quantity in Hospitalized Patients With Melatonin: A Quality Improvement Project at HCA (Hospital Corporation of America) Oak Hill Hospital

**DOI:** 10.7759/cureus.100510

**Published:** 2025-12-31

**Authors:** Tong Ren, Alisher Hamidullah, Sophia Samaranayake, Alexander Answine, Salman Muddassir, Rahul Mhaskar, Olu Oyesanmi, Mohamad Eid

**Affiliations:** 1 Division of Hematology and Medical Oncology, Beth Israel Deaconess Medical Center (BIDMC), Boston, USA; 2 Harvard Medical Faculty Physicians, Harvard Medical School, Boston, USA; 3 Internal Medicine, Oak Hill Hospital, Brooksville, USA; 4 HCA (Hospital Corporation of America) Healthcare/University of South Florida Morsani College of Medicine GME (Graduate Medical Education), Oak Hill Hospital, Brooksville, USA; 5 Internal Medicine, University of South Florida Morsani College of Medicine, Tampa, USA; 6 Medical Education, University of South Florida Morsani College of Medicine, Tampa, USA

**Keywords:** clinical sleep interventions, hospital patient well-being, melatonin, sleep latency reduction, sleep quality

## Abstract

This study investigated the effectiveness of melatonin in improving sleep quality among hospitalized patients. Researchers assessed sleep parameters, including duration, latency (time to fall asleep), awakenings, and subjective quality, before and after melatonin administration. Results showed significant improvement after melatonin across all measures, with increased sleep duration, faster sleep onset, fewer awakenings, and better reported sleep quality. These findings suggest melatonin supplementation as a potential strategy to enhance sleep in hospitalized patients.

## Introduction

Sleep, a fundamental pillar of human health, is essential for physical and cognitive restoration [1,2]. However, hospitalized patients often experience significant sleep disturbances due to various factors inherent to the hospital environment and the physiological effects of their illnesses. These disruptions include noise from medical equipment, frequent nighttime checks, unfamiliar environments, pain, and anxiety [3]. Such disturbances can lead to poor sleep quality, negatively impacting overall well-being and clinical outcomes [4].

Insufficient sleep in hospitalized patients has been associated with a cascade of adverse effects, including impaired immune function, increased risk of infections, prolonged hospital stays, and decreased patient satisfaction [5,6]. Additionally, sleep deprivation can impair cognitive function, making it more difficult for patients to participate actively in their care and recovery [7]. Restorative sleep is essential for mental well-being, and inadequate sleep has been linked to higher rates of delirium and anxiety in hospitalized patients. Therefore, improving sleep quality in hospitalized patients is crucial for optimizing healing and overall well-being.

While non-pharmacological interventions, such as maintaining regular sleep schedules and creating a calming environment, are typically the first-line approach [8], they may not always suffice, particularly for patients experiencing severe sleep disturbances or those with pre-existing sleep disorders [9]. This has led to an exploration of pharmacological solutions, including melatonin.

Melatonin, a naturally occurring hormone that regulates sleep-wake cycles, has emerged as a potential therapeutic option for promoting sleep in hospitalized patients [10]. It is well-tolerated and has minimal side effects when used at recommended doses [11]. Previous studies have suggested that melatonin supplementation may improve sleep quality and duration in various patient populations [12]. Despite these promising findings, the effectiveness of melatonin in hospitalized patients is not consistently demonstrated across all studies, and there is a need for further investigation into its efficacy in this specific setting.

Given the critical importance of sleep for patient recovery and the mixed results from prior research, our study aims to evaluate the effectiveness of melatonin in improving sleep quality and quantity among hospitalized patients. By addressing this gap in the literature, we hope to identify a feasible intervention that can be widely implemented to enhance patient outcomes during hospital stays. Improved sleep may also play a meaningful role in enhancing the quality of life for geriatric patients and those in post-acute or nursing home settings, where sleep disruption is particularly prevalent.

This study was previously presented as a meeting abstract at the 2024 Sleep Research Society Annual Meeting on June 3, 2024.

## Materials and methods

We conducted a before-and-after quality improvement study at HCA Florida Oak Hill Hospital in Brooksville, FL. The study was carried out across multiple inpatient units, including the medical-surgical unit, progressive care unit (PCU), and intensive care unit (ICU). Data collection took place over a seven-month period, from April 1, 2023, through October 31, 2023.

A total of 117 participants were included, with sleep-related parameters assessed before and after melatonin administration. Sleep outcomes were collected using a brief survey developed for this quality improvement project. The survey (Appendix) was not a full reproduction of the Richards-Campbell Sleep Questionnaire (RCSQ) or the Pittsburgh Sleep Quality Index (PSQI); instead, only specific conceptual items - sleep latency, number of awakenings, total sleep duration, and overall sleep quality - were adapted from these instruments and converted into a simplified 0-10 numeric rating format for internal use. No copyrighted wording, visual analog scales, or proprietary items from the original RCSQ or PSQI were used. The RCSQ and PSQI are cited for conceptual reference only [13,14]. Because no copyrighted content was reproduced, formal permission was not required.

Descriptive statistics were used to summarize baseline demographic and clinical characteristics. Continuous variables were reported as mean ± standard deviation (SD) or median with interquartile range (IQR), based on their distribution. Categorical variables were summarized as frequencies and percentages (n, %). To evaluate the impact of the intervention, pre- and post-melatonin administration sleep parameters were compared using the Wilcoxon signed-rank test. This non-parametric test was selected for its appropriateness in analyzing paired ordinal data and continuous data that did not meet assumptions of normality. The median difference between paired measurements was calculated, and statistical significance was set a priori at a two-sided p <0.05. All analyses were conducted using a complete-case approach, whereby subjects with missing data for a given variable were excluded from that specific analysis. Statistical analyses were performed using Stata version 17 (StataCorp LLC, College Station, TX). The study was not powered to conduct subgroup analyses.

In compliance with ethical guidelines, this study was granted an exemption from Institutional Review Board (IRB) oversight (IRB exemption reference number: 2024-1251). Melatonin is an FDA-approved medication for improving sleep, which contributed to the exemption determination.

Inclusion criteria

All adult patients (≥18 years) admitted to HCA Florida Oak Hill Hospital between April 1, 2023, and October 31, 2023, were eligible if they were hospitalized on the medical-surgical unit, PCU, or ICU and received melatonin as part of the standardized sleep-promotion protocol. Participants were required to have completed both the pre- and post-intervention sleep quality surveys.

Exclusion criteria

Patients were excluded if they were younger than 18 years, declined participation, had incomplete survey data, were admitted for less than 24 hours, were pregnant, were unable to take oral medications, or were receiving concurrent investigational sleep agents. Patients with cognitive impairment that precluded valid survey responses were also excluded.

## Results

The study analyzed a total of 117 participants, examining the impact of melatonin on various sleep parameters as well as demographic factors such as age, race, and gender. Ages ranged from 21 to 93 years, with a median age of 64 years. The largest portion of participants fell between 57 and 80 years old (56.4%, 66/117), representing a predominantly middle-aged to older adult population. Most participants identified as White (88.0%, 103/117), followed by African American (5.1%, 6/117), Hispanic (5.1%, 6/117), Asian (0.9%, 1/117), and Other (0.9%, 1/117). The sample included 53.0% women (62/117) and 47.0% men (55/117), reflecting a nearly even gender distribution. Baseline demographic information is summarized in Table 1.

**Table 1:** Demographic Data Baseline demographic characteristics of hospitalized patients included in the study (n = 117).

Characteristic	n (%)
Age (years)	
21-40	14 (12.0%)
41-56	21 (18.0%)
57-80	66 (56.4%)
>80	16 (13.7%)
Race/Ethnicity	
White	103 (88.0%)
African American	6 (5.1%)
Hispanic	6 (5.1%)
Asian	1 (0.9%)
Other	1 (0.9%)
Gender	
Female	62 (53.0%)
Male	55 (47.0%)
Total Sample Size	117 (100%)

**Figure 1:**
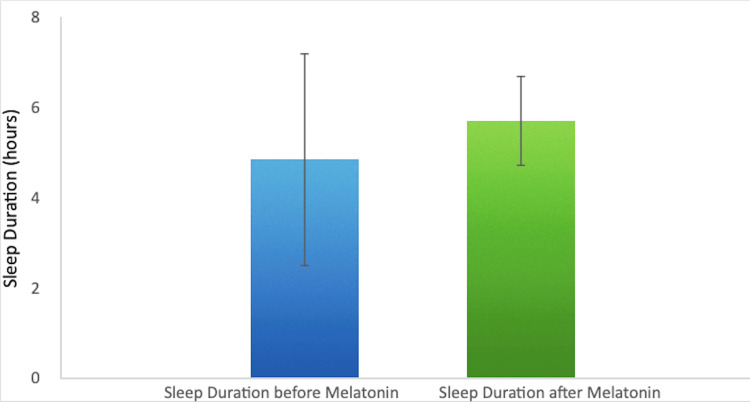
Improvement in Sleep Duration Following Melatonin Administration

Before melatonin administration, sleep disturbances were prevalent, with patients reporting an average sleep duration of 4.9 hours (Figure 1).

**Figure 2:**
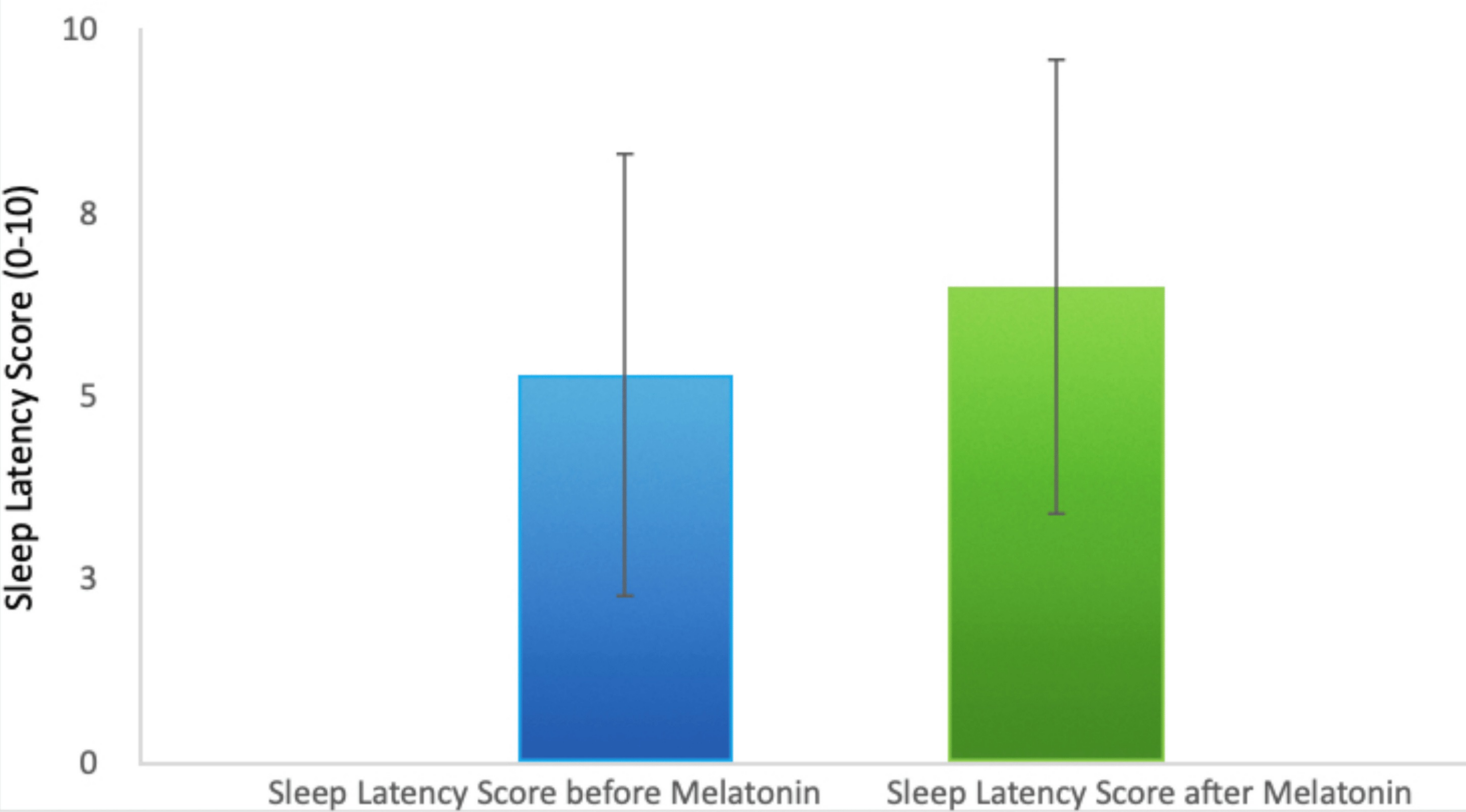
Change in Sleep Latency Before and After Melatonin

Melatonin intervention led to a significant improvement, increasing average sleep duration by 17.9% to 5.7 hours. Sleep latency, or the time taken to fall asleep, improved by 22.3%, indicating that patients fell asleep more quickly after melatonin administration (Figure 2).

**Figure 3:**
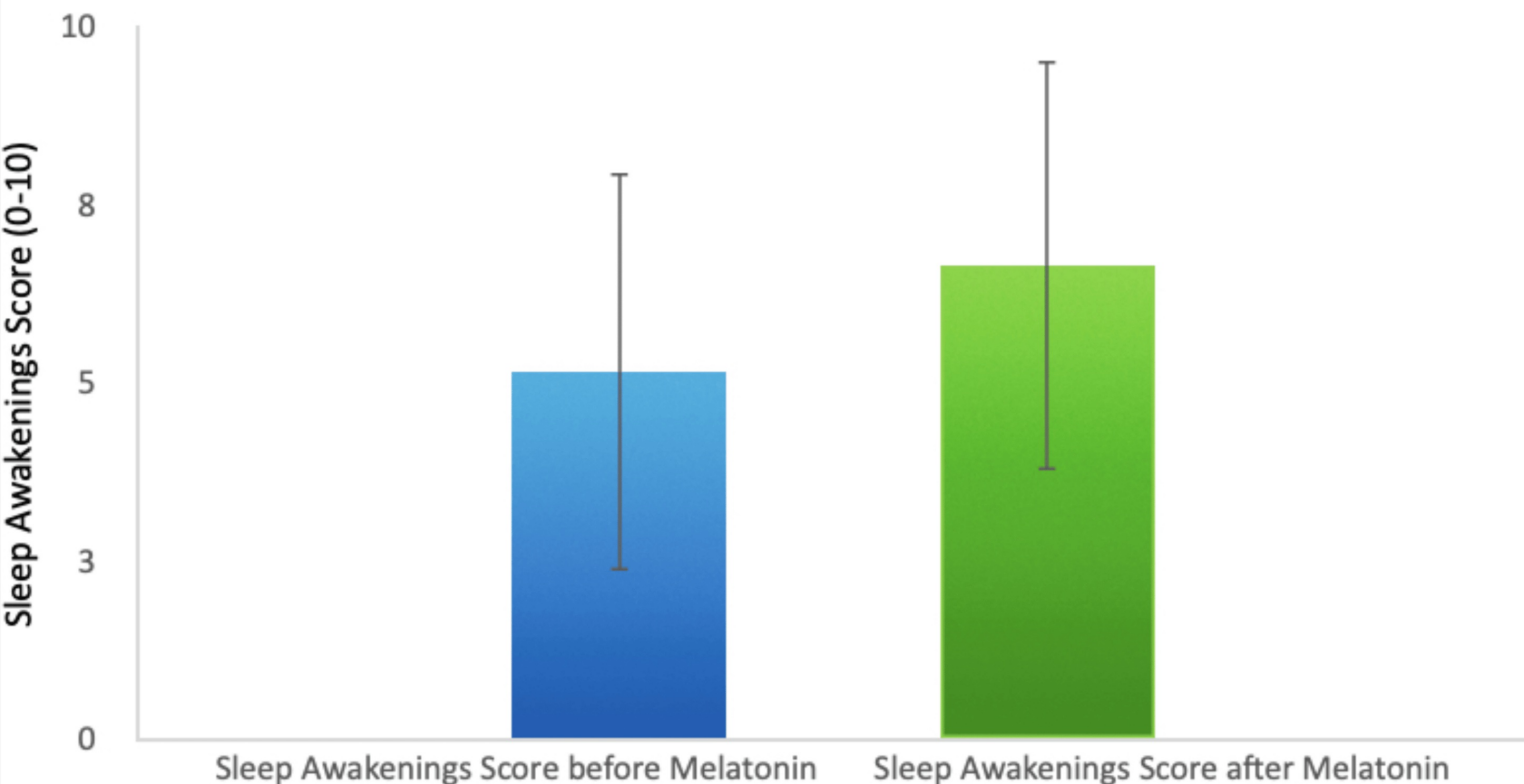
Reduction in Sleep Awakenings Post-Melatonin Administration

Additionally, the frequency of awakenings during the night decreased by 29.3%, which points to a reduction in sleep fragmentation (Figure 3).

**Figure 4:**
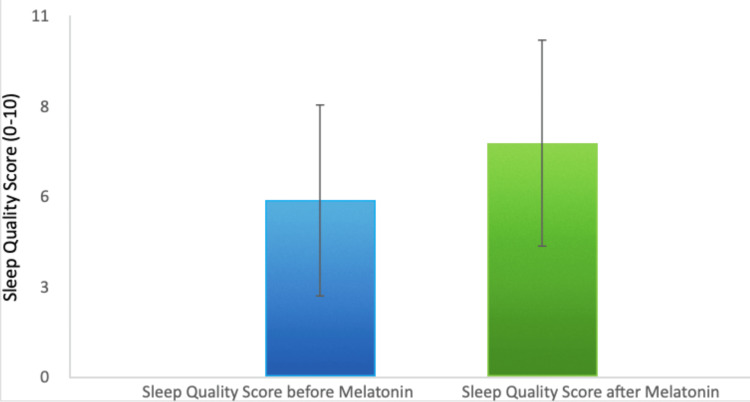
Improvement in Subjective Sleep Quality After Melatonin Administration

Finally, patients reported a 32% increase in overall sleep quality. The average sleep quality score increased from 5.41 to 7.15, highlighting melatonin's positive effect not only on the quantity but also on the subjective experience of sleep (Figure 4).

These results suggest that melatonin may serve as an effective intervention for improving both objective sleep metrics and patients' subjective perceptions of their sleep, contributing to enhanced recovery, reduced stress, and improved overall patient satisfaction during hospital stays.

## Discussion

Sleep disruption is a pervasive problem among hospitalized and critically ill patients, with consequences that include increased risk of delirium, prolonged recovery, and reduced quality of life. Melatonin, a hormone central to circadian rhythm regulation, has been investigated as a potential intervention to improve sleep quality and reduce delirium in these populations.

Recent systematic reviews and meta-analyses suggest that melatonin supplementation may improve subjective sleep quality and reduce delirium incidence, particularly in ICU and surgical patients. A 2025 meta-analysis of 32 randomized controlled trials found that melatonin may reduce delirium, slightly reduce ICU length of stay, and improve reported sleep quality, with a favorable safety profile. However, the certainty of evidence was limited by risk of bias and inconsistency, and effects on objective sleep parameters, anxiety, agitation, and mortality remain uncertain [15]. Another large meta-analysis confirmed a significant reduction in delirium incidence with melatonin or ramelteon, but this effect was most pronounced in surgical and ICU patients, with no significant benefit observed in medical inpatients [16].

Randomized controlled trials in ICU settings have demonstrated that melatonin can improve subjective sleep quality, as measured by validated questionnaires, but have not consistently shown reductions in delirium or improvements in objective sleep duration [17,18]. For example, Gandolfi et al. reported better sleep quality in the melatonin group compared to placebo, but no significant differences in delirium rates or sedative requirements [17]. Similarly, a Cochrane review concluded that the effects of melatonin on both subjective and objective sleep outcomes in ICU patients are uncertain due to limited and heterogeneous data [18].

In general medical inpatients, the evidence for melatonin is less robust. A narrative review highlights that while melatonin is safer than benzodiazepines and non-benzodiazepine hypnotics, especially in older adults, its efficacy for insomnia in hospitalized patients is not well established, and further research is needed [19]. A recent umbrella review of meta-analyses supports melatonin’s benefit for sleep-onset latency in patients with sleep disorders, but not for sleep efficiency, and calls for more high-quality trials in diverse inpatient populations [20].

Dose and timing of melatonin administration may influence its efficacy. A 2024 dose-response meta-analysis suggested that a dose of approximately 4 mg, taken about 3 hours before the desired bedtime, may optimize sleep-promoting effects [21]. Current guidelines, such as the 2023 American Thoracic Society statement, do not recommend routine pharmacologic treatment for sleep or circadian disruption in the ICU, citing mixed results and low certainty of evidence for melatonin and other agents [22]. Recent meta-analyses also indicate that melatonin does not significantly reduce ICU or hospital length of stay except in specific subgroups, such as post-cardiac surgery or COVID-19 patients [23].

However, most prior studies in hospitalized or ICU populations have used relatively low doses (2-5 mg), often without standardizing timing or environmental conditions, which may have contributed to inconsistent findings [24]. In contrast, our study utilized a higher dose of 9 mg, administered at bedtime in accordance with institutional sleep-promotion protocols. The rationale for using a higher dose was to enhance both sleep onset and maintenance in hospitalized patients, who often experience fragmented sleep and elevated stress levels compared with healthy individuals. Higher doses of melatonin have been reported to remain safe and well-tolerated in adults, with no significant adverse effects at doses up to 10 mg in short-term hospital settings. Thus, our study provides additional evidence supporting the potential benefit and tolerability of higher-dose melatonin in improving sleep quality and duration among inpatients.

Limitations

This study has several limitations. First, the sample size was relatively small (n = 117), which may limit the generalizability of the findings. Second, sleep parameters were evaluated using self-reported questionnaires, which are subjective and may introduce recall or response bias. Objective measures such as actigraphy or polysomnography were not utilized. Third, the study was conducted at a single community hospital, which may limit external validity and applicability to other healthcare settings. Finally, the analysis focused on short-term outcomes during hospitalization; long-term effects of melatonin use following discharge were not assessed.

## Conclusions

Melatonin administration resulted in significant improvements in sleep duration, latency, awakenings, and overall sleep quality in a diverse group of hospitalized patients. These findings suggest that melatonin may be a promising intervention for addressing sleep disturbances in the hospital setting, contributing to better patient outcomes by promoting restful sleep, enhancing recovery, and reducing stress and anxiety. Given the simplicity and safety profile of melatonin, it presents an attractive option for improving patient care. Further research is needed to explore the long-term effects of melatonin and to optimize dosing strategies for different patient populations.

## Appendices

**Table 2:** Sleep Assessment Survey

Sleep Assessment Item	Scale/Response Format	Scoring Interpretation
Sleep Latency	0-10 Numeric Rating Scale	0 = Unable to fall asleep at all; 10 = Fell asleep immediately
Number of Awakenings	0-10 Numeric Rating Scale	0 = Awakened many times throughout the night; 10 = No awakenings
Total Sleep Duration	Hours (numeric entry)	Patient writes total hours slept
Overall Sleep Quality	0-10 Numeric Rating Scale	0 = Very poor sleep quality; 10 = Excellent sleep quality
